# New Spectroelectrochemical Insights into Manganese and Rhenium Bipyridine Complexes as Catalysts for the Electrochemical Reduction of Carbon Dioxide

**DOI:** 10.3390/molecules28227535

**Published:** 2023-11-10

**Authors:** Alice Barbero, Laura Rotundo, Chiara Reviglio, Roberto Gobetto, Romana Sokolova, Jan Fiedler, Carlo Nervi

**Affiliations:** 1 Department of Chemistry, University of Torino, Via P. Giuria 7, 10125 Torino, Italy; 2 CIRCC (Interuniversitary Consortium of Chemical Reactivity and Catalysis), Via Celso Ulpiani 27, 70126 Bari, Italy; 3 Chemistry Division Brookhaven National Laboratory, Upton, NY 11973-5000, USA; 4 J. Heyrovský Institute of Physical Chemistry of the Czech Academy of Sciences, Dolejškova 3, 18223 Prague, Czech Republic

**Keywords:** CO_2_, spectroelectrochemistry, manganese, rhenium, carbon dioxide reduction

## Abstract

This study aimed to demonstrate the behavior of different complexes using IR spectroelectrochemistry (SEC), a technique that combines IR spectroscopy with electrochemistry. Four different Mn and Re catalysts for electrochemical CO_2_ reduction were studied in dry acetonitrile. In the case of Mn(apbpy)(CO)_3_Br (apbpy = 4(4-aminophenyl)-2,2′-bipyridine), SEC suggested that a very slow catalytic reduction of CO_2_ also occurs in acetonitrile in the absence of proton donors, but at rather negative potentials. In contrast, the corresponding Re(apbpy)(CO)_3_Br clearly demonstrated slow catalytic conversion at the first reduction potential. Switching to saturated CO_2_ solutions in a mixture of acetonitrile and 5% water as a proton donor, the SEC of Mn(apbpy)(CO)_3_Br displayed a faster catalytic behavior.

## 1. Introduction

The constant increase in CO_2_ concentration in the atmosphere and the depletion of fossil fuels have raised serious worldwide environmental and energy-related concerns [1]. Thus, one of the most prominent contemporary research topics is the conversion of CO_2_ into fuels using green, eco-friendly approaches and sustainable methodologies. Solar energy is a perfect engine that fits these requirements for driving the chemical conversion of CO_2_ (employing it as an energy vector in a carbon-based cycle), to be used as a raw starting material for fuel production [2]. Although direct artificial photosynthesis is conceptually attractive [3,4], its real-world application is still not optimal, and parallel approaches are actively being explored. An instantaneous and clean way of capturing the sun’s power is its direct conversion into electric energy using photovoltaic cells. The possible exploitation of this energy for the electrochemical reduction of CO_2_ into chemicals with higher energy content has attracted several researchers, resulting in the exponential growth of the number of papers that have lately appeared in this field.

The present contribution illustrates recent results of the spectroelectrochemical technique applied to the study of CO_2_ reduction using selected Mn and Re organometallic complexes. Cyclic voltammetry (CV) is a relatively rapid technique (one can envisage that the scan rate dictates the time scale of the experiments, which usually last a fraction of a second), whereas SEC is a slower technique (with a time scale in the order of minutes); however, the production of reduced/oxidized species in higher quantities enables the recording of their spectroscopic properties. SEC has been proven to be a very versatile technique, tested in different conditions and with different substrates and catalysts, assisting, in certain circumstances, with the interpretation of reduction mechanisms and the identification of intermediates. For example, in 2012, SEC was used to investigate CO_2_ reduction on a gold surface, making it possible to propose a reduction mechanism for CO_2_ in working conditions [5]. SEC has also been used several times to determine the mechanism of carbon dioxide reduction with different complexes and different metals. With Ru metal used for molecular and cluster catalysts, SEC is a fundamental technique employed for observing CO_2_ reduction products (CO, CO_3_^2−^, and HCOO^−^), as well as another two not fully identified carboxylate species [6,7]. The literature also reports examples of catalysts containing Co and Ni metals complexes, in which SEC has been used to identify intermediates, with interesting results [8,9]. Similarly, for complexes based on Mn and Re and with bipyridine-type structures, such as those used in this study, SEC has been used for discovering both radical intermediates and CO_2_ reduction products, in agreement with those previously reported [10,11].

**Scheme 1:**
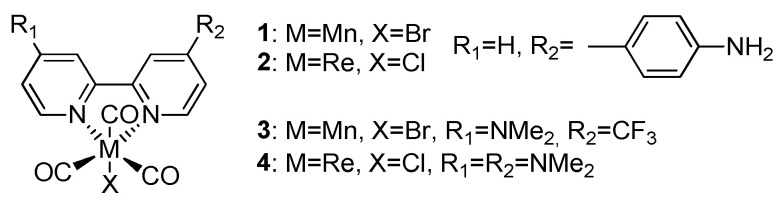
Sketch of the complexes under study.

We explored the activities of Re and Mn catalysts bearing bipyridine-like (bpy) units towards the electrochemical and photochemical reduction of CO_2_ [1,12,13,14]. Complexes **1**–**4** (Scheme 1) were synthesized here, following a previously reported procedure [15,16]; their electrochemical behavior is herein described in different conditions, as are their SEC properties and new insights into the overall mechanism. Cyclic voltammetry (CV) of Mn-bpy derivatives usually presents two subsequent chemically irreversible reductions [17], but only the second one, and only when in the presence of proton sources, is active toward CO_2_ conversion. Complex **1**, when chemically bonded to an electrode surface via 4(4-aminophenyl)-2,2′-bipyridine functionality (apbpy), shows extremely interesting catalytic properties in water, with a turnover number (TON) of up to 164,000 [12,13]. In contrast, Re-bpy complexes typically initially undergo a reversible reduction followed by a second irreversible and catalytically active reduction, even in the absence of proton donors. In very rare cases (and in kinetically much slower systems), Re-bpy derivatives can exhibit catalytic activities even at the first reduction peak, in a so-called slow regime [18]. We herein demonstrate via SEC that Re complex **2** is catalytically active at the initial reduction. SEC experiments were employed as a powerful tool to elucidate the mechanism details of CO_2_ reduction [10,19,20] in the case of analogous Mn and Re derivatives [14,21,22,23,24]. The SEC experiments and properties of the complexes are herein studied in a homogeneous phase for the first time.

Complexes **3** and **4** of the present set were selected for comparison, considering the promising and relatively high TON values observed for the electrochemical conversion of CO_2_ in a homogeneous solution. Complex **3** possessed a push-and-pull substituent group on the bpy ligand, resulting in a net negligible shift in the reduction potential with respect to the Mn-bpy complex without substituents, whereas good catalytic activities were recorded in both the CV and constant potential electrolysis (CPE) experiments. Complex **4**, bearing a strong electron-donor ligand, showed a shift in reduction processes to more negative potentials, with improvements in the catalytic activity [16]. We selected these two different methodologies to enhance the catalytic behavior of the catalysts, and tested them in SEC experiments. The four complexes, which are promising catalysts [15,16] (see Table 1), were studied in comparative tests under SEC conditions.

## 2. Results

### 2.1. Mn(apbpy)(CO)_3_Br

**Figure 1:**
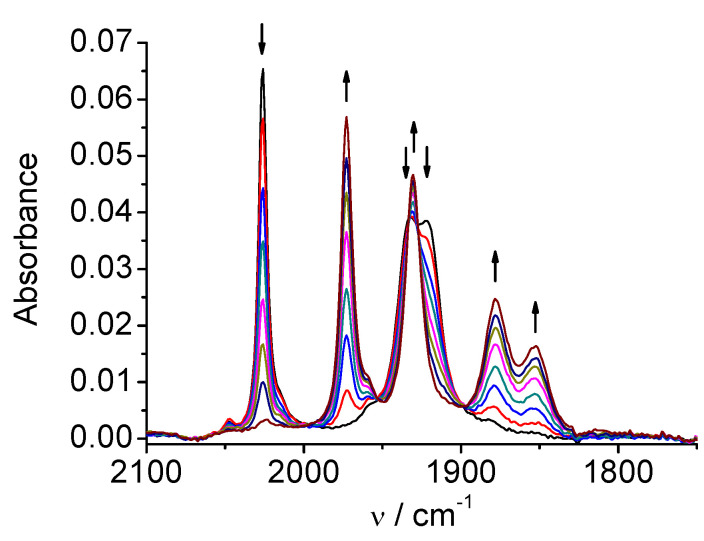
IR spectroelectrochemical response on the first reduction of **1** in MeCN/0.1 M Bu_4_NPF_6_. Arrows up and down represent increases and decreases in the absorption bands, respectively.

**Figure 2:**
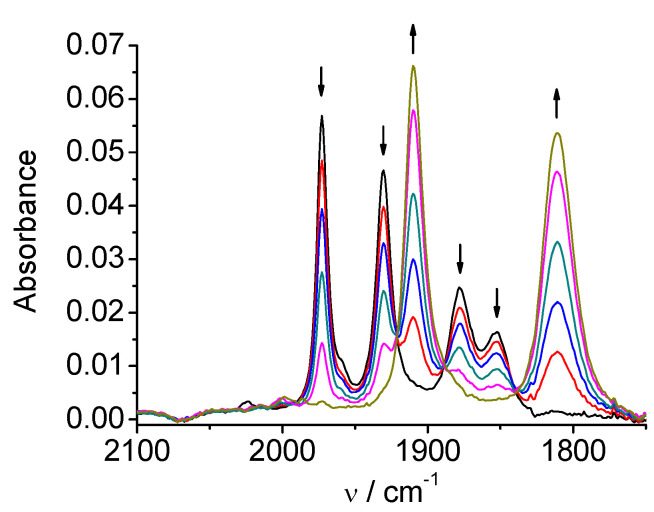
IR spectroelectrochemical response on the second reduction of **1** in MeCN/0.1 M Bu_4_NPF_6_.

**Scheme 2:**
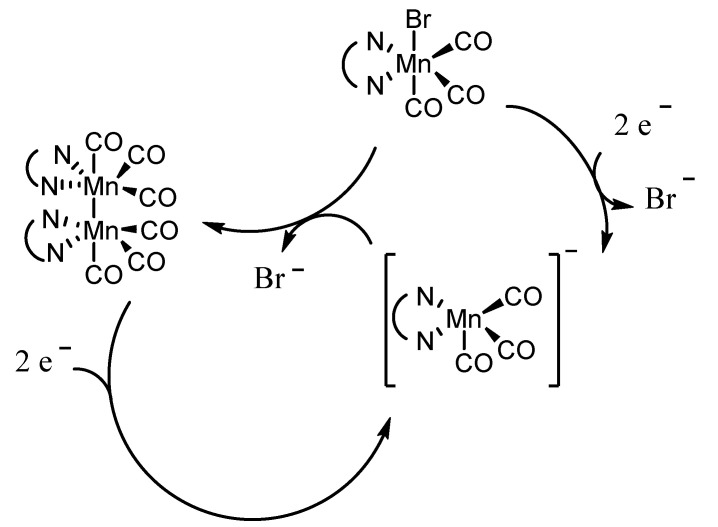
Summary of the overall mechanism of [Mn(bpy-type)(CO)_3_Br] complexes leading to the Mn anion, the active catalyst in electrochemical CO_2_ reduction.

CV of Mn(apbpy)(CO)_3_Br (**1**) in dry acetonitrile (MeCN) displayed two chemically irreversible reductions at E_p_ = −1.21 V and E_p_ = −1.41 V vs. Ag/AgCl [15]. In situ SEC spectra of **1** (Figure 1) at the first reduction potential showed conversion of the original ν(CO) bands (2026, 1933, and 1922 cm^−1^) into four ν(CO) bands (1973, 1931, 1878, and 1852 cm^−1^). This behavior is typical for the formation of a Mn–Mn dimer, which has recently been demonstrated by Daasbjerg [23] to proceed, for the similar Mn(bpy)(CO)_3_Br complex, via its 2e^−^ reduction involving an extremely rapid Br^‾^ release, and the subsequent rapid chemical reaction between the penta-coordinated anion and the neutral Mn complex. The second reduction of **1**, which occurred at more negative potentials, split the dimer [10,25,26,27,28], leading to the monomeric penta-coordinated anion [Mn(apbpy)(CO)_3_]^−^ This anion exhibits two ν(CO) bands, at 1909 and 1811 cm^−1^ (two wider, almost degenerate vibration modes) (Figure 2). The overall mechanism is briefly summarized in Scheme 2.

No significant difference was observed when the SEC experiments (first and second reduction) were performed with the solution saturated with carbon dioxide. Only when the reduction potential was changed to more negative values, behind the second reduction peak of **1**, did the band of dissolved CO_2_ at 2342 cm^−1^ decrease (Figure S1) and bands at 1684 and 1643 cm^−1^ increase. These two bands are attributed to the formation of the HCO_3_^−^/CO_3_^2−^ system that usually appears after CO_2_ reduction [10,29]. Although we cannot completely exclude a direct reduction in CO_2_, the potential applied (−1.75 V vs. Ag/AgCl) is not sufficiently negative to reduce pure CO_2_-saturated solution in dry acetonitrile [15]; therefore, these observations suggest a slow catalytic process not evident in faster CV experiments. To the best of our knowledge, this is the first attempt to perform reduction and SEC procedures on Mn complexes at this negative potential in the absence of proton donors. In the relatively short SEC process time (albeit much longer with respect to CV), it is possible to assert that the two bands at 1909 and 1811 cm^−1^ partially lose intensity, but at the same time, the bands at 1684 and 1643 cm^−1^ sharply increase (Figure S1), which can be interpreted by a slow catalytic process, with probable catalyst decomposition over a longer time and at such negative potentials.

**Figure 3:**
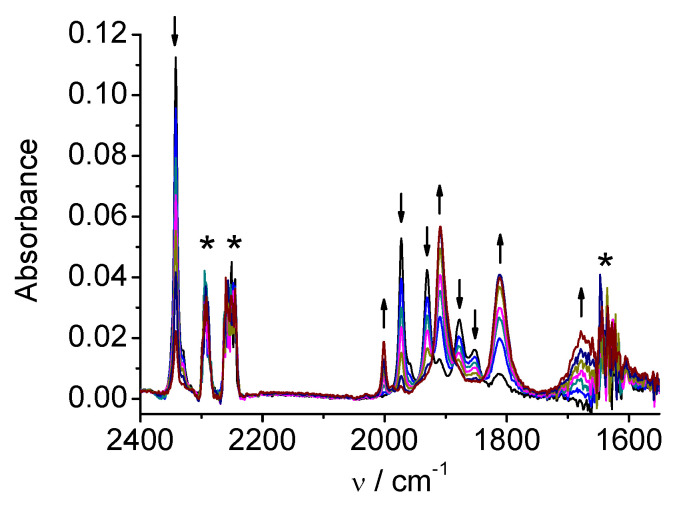
IR spectroelectrochemical response on the second reduction of **1** in MeCN/0.1 M Bu_4_NPF_6_/5% H_2_O under CO_2_ (*: distorted due to strong absorption of the MeCN/water mixture as a solvent).

SEC performed at the second reduction peak (i.e., −1.41 V), but employing acetonitrile solution containing 5% of water, exhibited typical catalytic CO_2_ reduction (Figure 3), a decrease in the CO_2_ absorption band at 2343 cm^−1^, and increases in bands of HCO_3_^−^/CO_3_^2−^ at 1684 and 1643 cm^−1^. Other bands showed a trend similar to that presented in Figure 2, i.e., the reduction of the dimer [Mn(apbpy)(CO)_3_]_2_ into a monomeric anion. These results agree with previous observations from cyclic voltammetry and exhaustive electrolysis experiments [15], demonstrating electrocatalysis in the presence of proton donors.

### 2.2. Re(apbpy)(CO)_3_Cl

**Figure 4:**
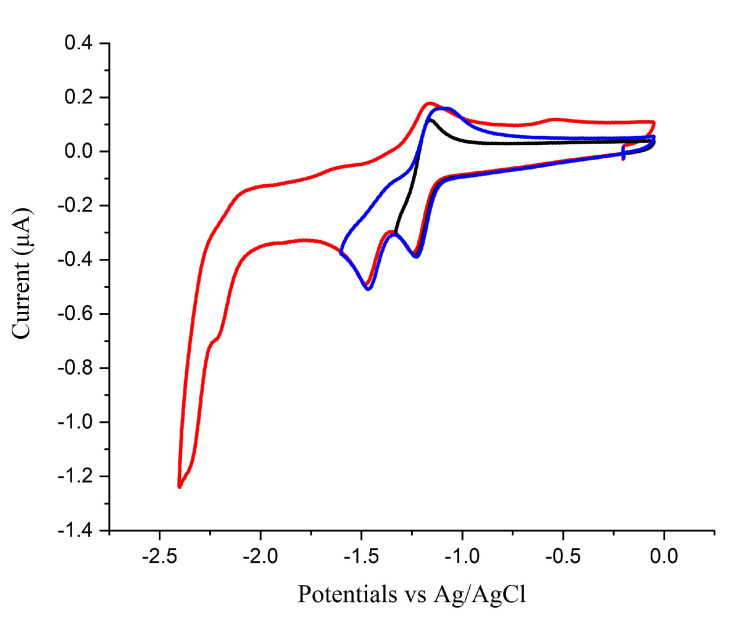
Cyclic voltammetry of **2** in MeCN/0.1 M TBAPF_6_ under Ar.

CV of the rhenium complex **2** (Figure 4) showed an initial reversible reduction at E_1/2_ = −1.28 V, and a second irreversible reduction at E_p_ = −1.74 V vs. Ag/AgCl. SEC of **2** showed that the reduction mechanism significantly differed from that observed for Mn analogue **1**.

**Figure 5:**
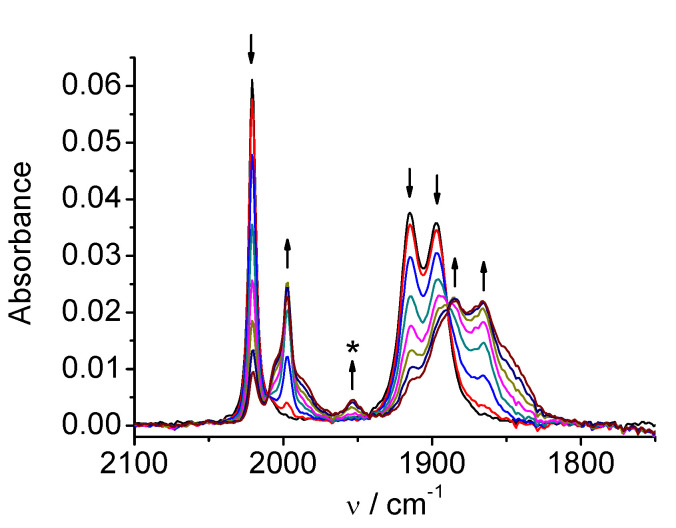
IR spectroelectrochemical response on the first reduction of **2** in MeCN/0.1 M Bu_4_NPF_6_ (*: unidentified side-product).

**Figure 6:**
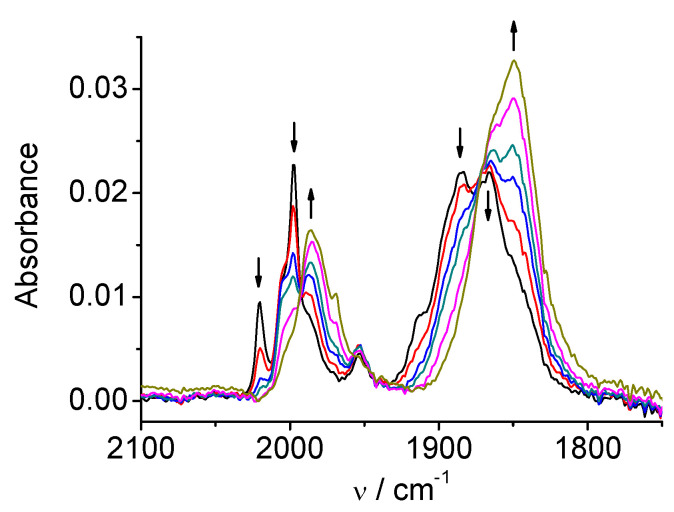
IR spectroelectrochemical response on the second reduction of **2** in MeCN/0.1 M Bu_4_NPF_6_.

On the reduction at the first peak (Figure 5), the original three ν(CO) bands (2021, 1915, and 1897 cm^−1^) shifted to lower wavenumbers (1997, 1886, and 1866 cm^−1^), but the characteristics of the three-band spectrum, and consequently, the coordination symmetry, remained unchanged. Only a low-energy shift in the CO bands due to the electrostatic effect of the electron uptake is evident in Figure 5, indicating quantitative conversion of the neutral species into the chemically stable radical anion. Hence, the single-electron reduction product remained hexacoordinated, and no dimerization took place, in contrast to Mn complex **1**. After the electrolysis at the first peak (Figure 5), the bands of the double-electron reduction product also appeared, due to the close potentials of the first and second reduction peaks. Development of the bands of the double-electron reduction product continued during reduction at the second peak (Figure 6); as a result, the spectrum consisted of two main bands at 1986 cm^−1^ and 1850 cm^−1^ (two wider, almost degenerate vibrations), corresponding to the anion [Re(apbpy)(CO)_3_]^‾^.

**Figure 7:**
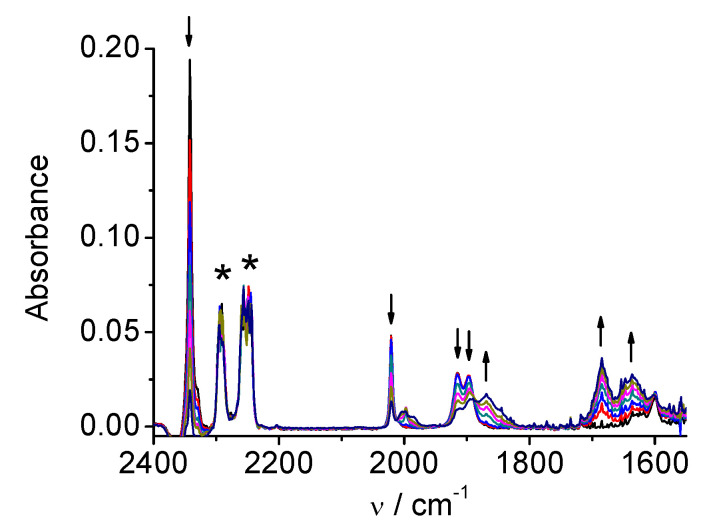
IR spectroelectrochemical response on the first reduction of **2** in MeCN/0.1 M Bu_4_NPF_6_ under CO_2_ (*: artefact due to strong absorption of solvent).

SEC experiments performed under CO_2_ revealed the catalytic activity of complex **2**. Figure 7 clearly shows that carbon dioxide is catalytically reduced at potentials of the first reduction peak of the complex. The decrease in the CO_2_ concentration in the solution (band at 2342 cm^−1^) and the formation of the products (HCO_3_^−^/CO_3_^2−^, bands around 1685 and 1645 cm^−1^) take place simultaneously with the reductive transition of **2** to **2**^‾^. The results thus show that although **2**^‾^ does not lose a Cl atom to form the penta-coordinated complex in the CV time scale, the coordination bond is labilized, and the halide can be substituted by solvent or substrate (CO_2_). The labilized coordination site plays a key role in the catalytic mechanism. Indications that the catalytically active complex does not necessarily need to be formed through the second reduction of a bipyridine-tricarbonyl complex have previously been posited for similar complexes [14]. Complex **2** is a clear example, demonstrated by a relatively rapid technique such as SEC, that exhibits catalytic activity when reduced only at the initial single-electron step.

The metal–halogen bond (M–X) is much weaker in Mn complex **1** than in Re complex **2**. For example, whereas complex **1** does not normally release Br^-^ in MeCN solution at r.t., rapid solvolysis of the Mn–Br bond in MeCN has been observed in the Mn complex carrying a bpy ligand modified with a hydroxyphenyl moiety [21], even at open circuit potentials.

**Figure 8:**
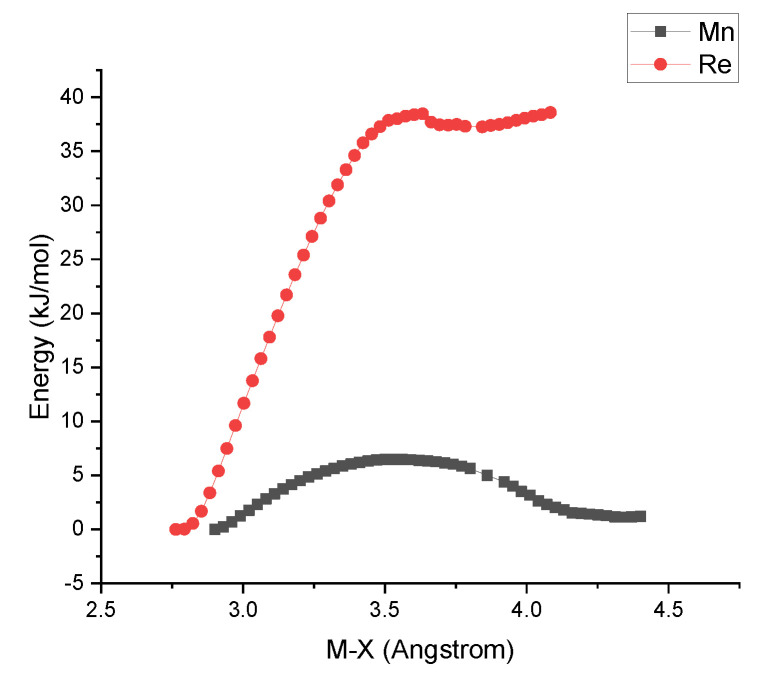
Relaxed geometry scan for **1**^‾^ (black squares) and **2**^‾^ (red circles) as a function of M–X distances in acetonitrile as the solvent.

The kinetics of the halogen-releasing process are greatly enhanced in Mn complexes whenever the corresponding reduced species are considered, accounting for the chemically irreversible and the reversible first reduction steps of **1** and **2**, respectively. DFT relaxed geometry scans of **1^‾^** and **2^‾^** as a function of M–X distances (Figure 8) clearly show that the radical anion Mn complex **1^‾^** has a much higher propensity to release the halogen than the Re counterpart **2^‾^**. This is confirmed by the fact that the Mn–Br distance of the optimized geometry **1^‾^** is 0.294 Å longer than that of **1**, while the Re–Cl bond of **2^−^** is only 0.052 Å longer than that of **2** (see the Supplementary Materials for details).

### 2.3. Mn(bpy-4,-4′-CF_3_-NMe_2_)(CO)_3_Br

**Figure 9:**
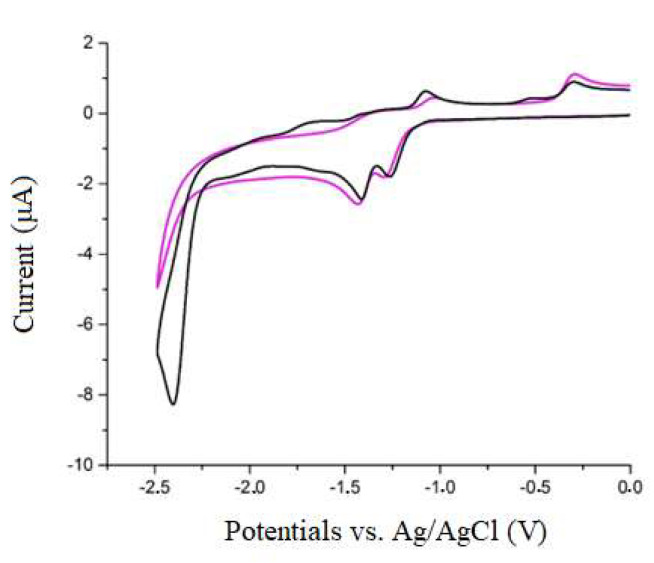
Cyclic voltammetry of **3** in MeCN/0.1 M TBAPF_6_ under Ar (violet line) and under CO_2_ (black line).

CV of Mn complex **3** (Figure 9) consisted of two chemically irreversible reductions at E_p_ = −1.27 and E_p_ = −1.41 V vs. Ag/AgCl. SEC of **3** shows similar results to those observed for **1**.

**Figure 10:**
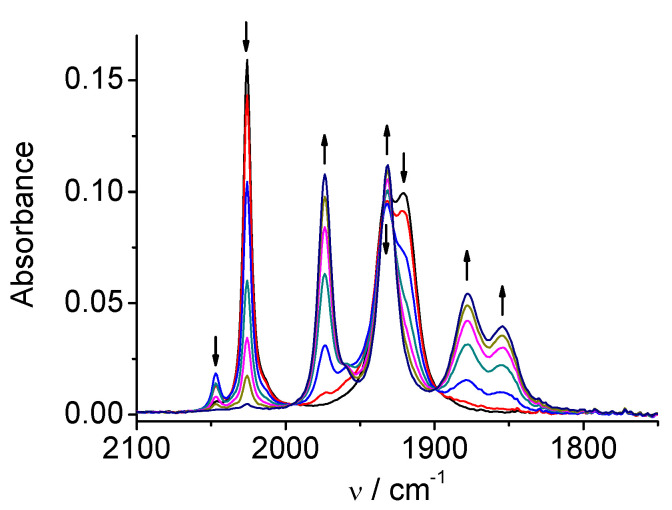
IR spectroelectrochemical response on the first reduction of **3** in MeCN/0.1 M Bu_4_NPF_6_.

**Figure 11:**
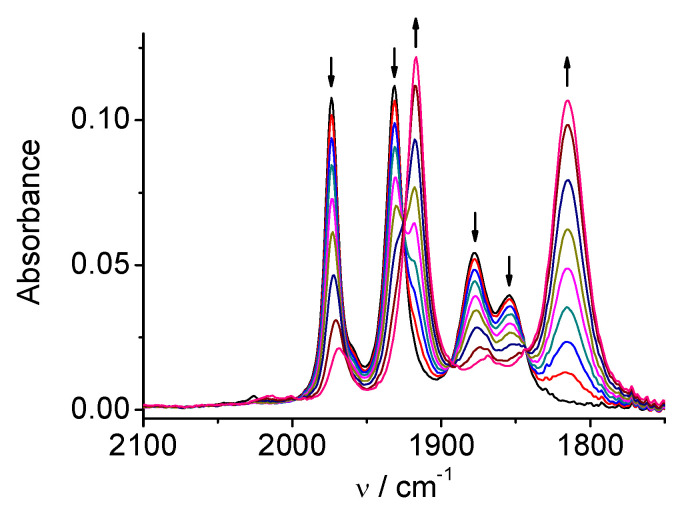
IR spectroelectrochemical response on the second reduction of **3** in MeCN/0.1 M Bu_4_NPF_6_.

The three original ν(CO) bands (2026, 1932, and 1921 cm^−1^) were converted at the first single-electron reduction (Figure 10) into four bands of the dimer (1974, 1932, 1878, and 1854 cm^−1^). The coincidence of the bands of the original complex and of the single-electron reduction product at 1932 cm^−1^ caused a poorly distinguishable spectral change in this region. The second reduction (Figure 11) led to the anionic species [Mn(bpy-4,4′-CF_3_-NMe_2_)(CO)_3_]^‾^ with the characteristic two-band spectrum (1917, 1816 (wide) cm^−1^) in the carbonyl region. A small band at 2047 cm^−1^ appeared as transient, and could be attributed to solvent-substituted species [Mn(bpy-4,4′-CF_3_-NMe_2_)(CO)_3_(MeCN)]^+^, whose formation can be facilitated due to an electron-transfer catalytic (ETC) effect, as observed in similar complexes [21].

SEC experiments with carbon dioxide-saturated solution showed that no CO_2_ reduction takes place during electrolysis at potentials of the first reduction wave, and the spectral change (Figure S2) resembles that under argon. Carbon dioxide reduction starts only at the potential of the second reduction peak (Figure S5, see bands HCO_3_^−^/CO_3_^2−^ at 1685 and 1647 cm^−1^), similarly to that observed for compound **1**.

### 2.4. Re(bpy-4,4′-NMe_2_)(CO)_3_Cl

**Figure 12:**
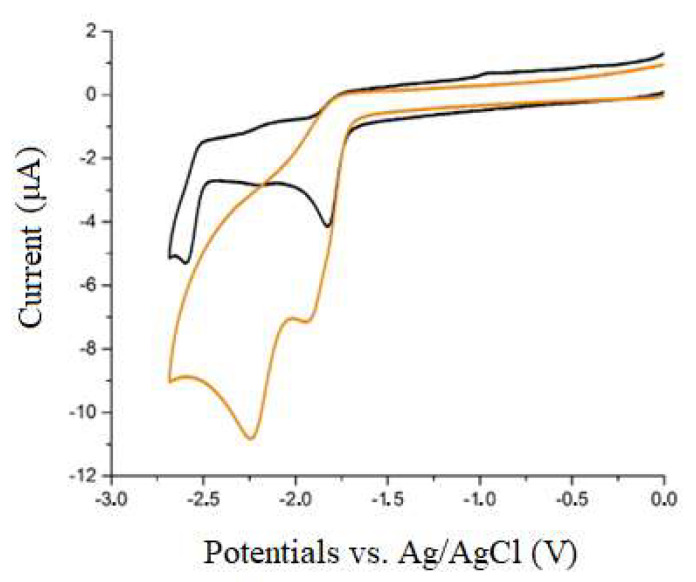
Cyclic voltammetry of **4** in MeCN/0.1 M TBAPF_6_ under Ar (black line) and under CO_2_ (orange line).

**Figure 13:**
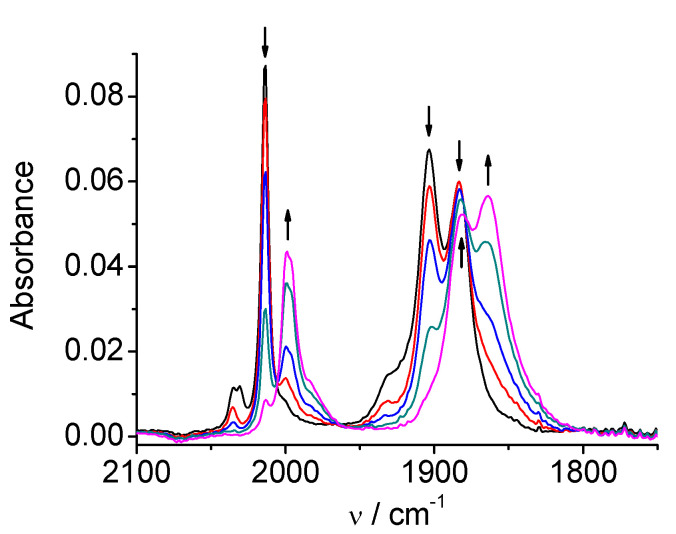
IR spectroelectrochemical response on the first reduction of **4** in MeCN/0.1 M Bu_4_NPF_6_.

**Figure 14:**
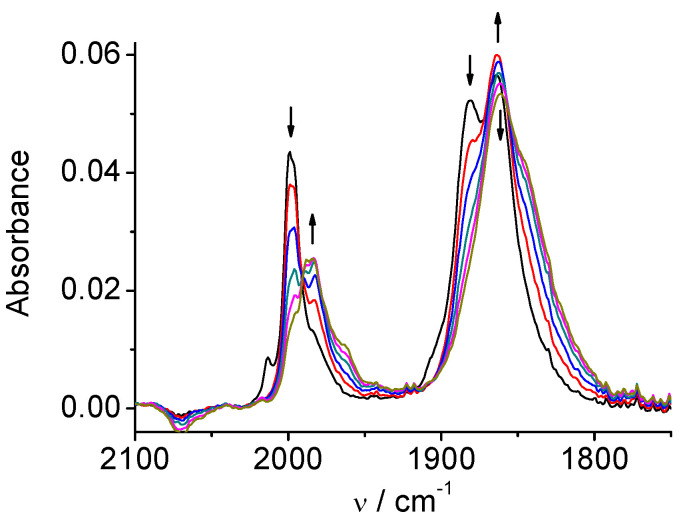
IR spectroelectrochemical response on the second reduction of **4** in MeCN/0.1 M Bu_4_NPF_6_.

CV of Re complex **4** (Figure 12) exhibited a single chemically irreversible double-electron reduction at E_p_ = −1.82 V, and another much more negative reduction (ligand-centered) at E_p_ = −2.60 V vs. Ag/AgCl. The two single-electron reduction steps were merged in the case of complex **4** into the one double-electron peak observed in CV [16]. However, a carefully controlled spectroelectrochemical reduction revealed that the reduction steps could be distinguished according to spectral changes. The first spectral change during the reduction process resembled that observed for single-electron reduction of complex **2**. Original carbonyl bands (2014, 1903, 1883 cm^−1^) were shifted to lower wavelengths (1998, 1982, 1864 cm^−1^, Figure 13). Continued reduction at only slightly more negative potential (two merged reduction steps) (Figure 14) produced an anion ([Re(bpy-4,4′-NMe_2_)(CO)_3_]^‾^) with the ν(CO) bands at 1986 and 1862 (wide) cm^−1^, similarly to what was observed for the analogous rhenium complex **2**.

**Figure 15:**
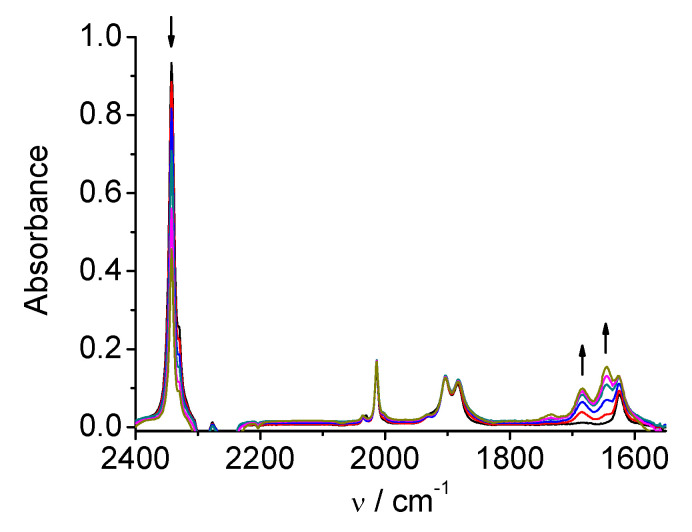
IR spectroelectrochemical response during the reduction of **4** in MeCN/0.1 M Bu_4_NPF_6_ under CO_2_.

In the presence of CO_2_, the CV peak current significantly increased, suggesting reactivity with the reduced species (orange curve in Figure 12). The spectroelectrochemical reduction of **4** in a solution saturated with CO_2_ produced bands at 1684 and 1646 cm^−1^ (HCO_3_^−^/CO_3_^2−^) simultaneously, with decreases in the CO_2_ band at 2342 cm^−1^. The original spectrum of the complex remained almost unchanged (Figure 15). This result indicated that a different SEC mechanism of electrocatalytic carbon dioxide reduction was observed: it began at the first double-electron reduction potential of complex **4**, and the catalytic cycle restored the original neutral complex **4**.

In order to prove the possible influence of a different solvent on the reduction mechanism, the SEC reductions of **1**–**4** were repeated using dimethylacetamide (DMA)/0.1 M Bu_4_NPF_6_ as a solvent. In the case of compounds **1**–**3**, these experiments did not reveal any essential differences in the mechanism of the reduction and generated reduced species (Figures S4–S9). However, the reduction of complex **4** in DMA directly proceeded in one double-electron step to the anion ([Re(bpy-4,4′-NMe_2_)(CO)_3_]^‾^ (Figure S10). The single-electron intermediate observed in the acetonitrile solution was not detected in the DMA solutions. The reason could have been a different substitution equilibrium chloride/solvent for the 1e^‾^ reduced intermediate, and consequently, a different intrinsic reduction mechanism (EEC vs. ECE). The principal ν(CO) frequencies observed experimentally from SEC in both solvents (acetonitrile and dimethylacetamide) are summarized in Table 2.

## 3. Discussion and Conclusions

IR spectroelectrochemistry has been used to highlight the catalytic behavior of four bpy complexes of Mn and Re. The CV of Mn complex **1** and that of Re complex **2** exhibited irreversible and reversible first chemical reductions, respectively. The corresponding SEC experiments clearly indicated that at the first reduction, **2** was already catalytically active in the CO_2_ conversion. Only at more negative potentials did **1** display an unprecedented very slow catalytic activity, which was greatly enhanced by the presence of 5% water as a proton donor. The higher lability of the M–X bond in **1**^‾^ with respect to **2**^‾^ has been interpreted using DFT calculations. Complexes **3** and **4** showed analogue catalytic activities, with the latter characterized by an overlapped 2e first reduction, partially resolved by SEC. In dimethylacetamide solutions, **4** was reduced by two electrons, and SEC could not discriminate the individual steps. These results highlight the suitability and effectiveness of SEC in investigating such catalytic processes, in a time scale slightly longer than that of CV.

## 4. Materials and Methods

**Synthesis.** The reagents were purchased from Sigma-Aldrich and used without further purification. The ligand 4-(4-aminophenyl)-2,2′-bipyridine was synthesized through a multistep reaction, following the slight modification of previously published procedures [30]. First, 4.1 g of 4-nitrobenzaldehyde was added to 50 mL of EtOH, and the mixture was heated at 70 °C until all the solid was dissolved. Subsequently, 3.1 g of sodium pyruvate dissolved in 15 mL of H_2_O was slowly added, and the mixture was cooled on ice. Then, 25 mL of NaOH 0.5 M was slowly added, and the mixture was left on ice for a further 2.5 h. HCl 2 M was added dropwise to the mixture until neutrality was achieved, and the solution was filtered. The solid, 4-(4-nitrophenyl)-2-oxo-3-butenoic acid, was washed with ethanol and air-dried. After that, 3.0 g of the acid, 4.4 g of pyridinium iodide, and 8.3 g of NH_4_OAc were added to 90 mL of water. The suspension was heated at reflux for 6 h. The resulting ammonium salt of 4-(4-nitrophenyl)-6-carboxylate-2,2′-bipyridine was washed with water and acetone, air-dried, and finally heated under vacuum until gas evolution (CO_2_) ceased and a black solid formed. The black solid was dissolved in EtOAc, activated charcoal was added, and the suspension was refluxed for 20 min. After this procedure, the solution was filtered over Celite, and the solvent was removed under vacuum. The dried yellowish solid, 4-(4-nitrophenyl)-2,2′-bipyridine, was dissolved in 30 mL of EtOH, mixed with 0.30 g of 10% Pd/C, 3.7 mL of hydrazine was added at 50%, and finally, the flask was heated at reflux for two hours. After a second addition of hydrazine (2 mL) and a further 30 min of refluxing, the liquid mixture was filtered over Celite; the solid was dissolved in CH_2_Cl_2_ and transferred into a separation funnel, and water was added. The organic solution was then dried through contact with anhydrous Na_2_SO_2_, and the solvent was removed under vacuum. The desired ligand, 4-(4-aminophenyl)-2,2′-bipyridine, was obtained with an overall yield of 40%. ^1^H-NMR: ((CD_3_)_2_CO) 8.71 (s, 1H), 8.70 (dm, 1H, J = 5.0 Hz), 8.60 (d, 1H, J = 7.9 Hz), 7.92 (td, 1H, J = 7.8 Hz, J = 1.8 Hz), 7.63 (d, 2H, J = 8.8 Hz), 7.60 (dd, 1H, J = 5.3 Hz, J = 2.0 Hz), 7.41 (ddd, 1H, J = 7.9 Hz, J = 5.0 Hz, J = 1.2 Hz), 6.83 (d, 2H, 8.8 Hz), 5.07 (s, 2H).

Mn complex **1** was obtained by mixing equimolar quantities of the ligand 4-(4-aminophenyl)-2,2′-bipyridine and the carbonyl complex Mn(CO)_5_Br in diethyl ether and refluxing for 4 h. The resulting yellow solid was washed with clean diethyl ether and left to dry under an inert atmosphere. Re complex **2** was obtained by mixing an equimolar amount of 4-(4-aminophenyl)-2,2′-bipyridine and Re(CO)_5_Cl in toluene and refluxing for 4 h. The resulting solid was first washed with clean toluene and then with petroleum ether and left to dry under an inert atmosphere. Re and Mn carbonyl complexes **3** and **4** were synthesized through a similar procedure by reacting the corresponding substituted bipyridines with the Re(CO)_5_Cl or Mn(CO)_5_Br precursors, following a similar synthetic approach as previously reported [15,16]. The ligand and the complexes were characterized using ^1^H NMR spectroscopy. NMR spectra were recorded with a JEOL EX 400 spectrometer (^1^H operating frequency 400 MHz) at 298 K; data were treated using Jeol Delta Software. ^1^H chemical shifts were relative to TMS (δ = 0 ppm) and referenced against solvent residual peaks.

**Cyclic Voltammetry.** Cyclic voltammetry experiments were performed using a 1 mM solution of the complexes under study with a Biologic 300SP potentiostat. A single-compartment cell was employed, equipped with a glassy carbon electrode (GCE, Ø = 1 mm) as the working electrode, a Pt counter electrode, and a Ag/AgCl (KCl 3 M) reference electrode. Acetonitrile (MeCN) and dimethylacetamide (DMA) solvents were freshly distilled over calcium hydride. Tetrabutylammonium hexafluorophosphate (Bu_4_NPF_6_, Sigma-Aldrich, Saint Louis, MI, USA, 98%) was recrystallized three times from ethanol solutions and used as the supporting electrolyte (0.1 M). Ar- and CO_2_-saturated conditions were achieved by purging gases for 5 min before the experiment.

**Spectroelectrochemistry.** Spectroelectrochemical experiments were performed using a Nicolet iS50 NIR-FTIR spectrometer equipped with an optically transparent thin-layer electrode (OTTLE) cell, employing Pt working and counter electrodes and a Ag wire as the pseudoreference electrode [31,32]. The spectra were collected during a slow (5 mV/s) voltammetric scan in the range of the voltammetric peaks. The increase in the applied potential was repeatedly interrupted for a while (ca. 10 s) when the individual spectra were collected.

**DFT Calculations.** Gaussian 09 Rev.D.01 [33] was used for computational studies. The conductor-like polarizable continuum model (CPCM) [34,35] with acetonitrile as a solvent was included to account for solvent effects. Geometry optimizations were carried out without any constraints using the B3LYP functional [36,37], the optimized def2-TZVP basis set for metals and halogens, and the def2-SVP basis set for all other atoms [38,39]. The D3 version of Grimme’s dispersion with the Becke–Johnson damping scheme [40] method was applied. Thermal corrections for entropy and enthalpy at 298 K to the electronic energies were calculated, and the natures of all stationary points were confirmed by normal-mode analysis (no imaginary frequencies were found). For radical anions, unrestricted Kohn–Sham formalism (UKS) was adopted.

## Figures and Tables

**Table 1:** Reduction potentials in MeCN solutions, potentials vs. Ag/AgCl, TONs, and the faradaic efficiency (FE) of the four complexes under study [15,16].

Complex	CPE (V)	E_p1_ (V)	Mixture	Time (min)	E_p2_ (V)	TON_CO_	FE_CO_%
**1**	−1.73	−1.51	4% water	90	−1.76	12	93
**2**	−2.00	−1.70	8% MeOH	120	−2.12	12	96.4
**3**	−1.50	−1.31	5% water	420	−1.42	26	84
**4**	−2.00	−1.82	5% MeOH	600	−1.82	31.5	100

**Table 2:** Carbonyl stretching frequencies (cm^−1^) from the SEC measurements.

Complex	Solvent	Native State	1e Reduction State	2e Reduction State
**1**	MeCN	2026, 1933, 1922	1973, 1931, 1878, 1852	1909, 1811
	DMA	2021, 1930, 1912	1969, 1927, 1875, 1853	1907, 1810
**2**	MeCN	2021, 1915, 1897	1997, 1886, 1866	1986, 1850
	DMA	2016, 1912, 1890	1992, 1879, 1860	1981, 1860
**3**	MeCN	2026, 1932, 1921	1974, 1932, 1878, 1854	1917, 1816
	DMA	2021, 1930, 1912	1972, 1929, 1877, 1855	1916, 1815
**4**	MeCN	2014, 1903, 1883	1998, 1882, 1864	1986, 1862
	DMA	2009, 1899, 1877	not observed	1980, 1859

## Data Availability

Not applicable.

## Supplementary Materials

The following supporting information can be downloaded at https://www.mdpi.com/article/10.3390/molecules28227535/s1, Figure S1: Spectroelectrochemistry of **1** in MeCN under CO_2_: reduction behind the second reduction peak (−2.1 V vs. Fc/Fc^+^); Figure S2: Spectroelectrochemistry of **3** in MeCN under CO_2_: first reduction; Figure S3: Spectroelectrochemistry of **3** in MeCN under CO_2_: second reduction; Figure S4: Spectroelectrochemistry of **1** in DMA: first reduction; Figure S5: Spectroelectrochemistry of **1** in DMA: second reduction; Figure S6: Spectroelectrochemistry of **2** in DMA: first reduction; Figure S7: Spectroelectrochemistry of **2** in DMA: second reduction; Figure S8: Spectroelectrochemistry of **3** in DMA: first reduction; Figure S9: Spectroelectrochemistry of **3** in DMA: second reduction; Figure S10: Spectroelectrochemistry of **4** in DMA: double-electron reduction; DFT optimized structures of **1**, **1**^‾^, **2**, and **2**^‾^. Figure S11: ^1^H NMR spectra of the ligand 4-(4-aminophenyl)-2,2′-bipyridine in (CD_3_)_2_CO.

## References

[1] Rotundo L., Gobetto R., Nervi C. (2021). Electrochemical CO_2_ reduction with earth-abundant metal catalysts. Curr. Opin. Green Sust. Chem..

[2] Aresta M., Dibenedetto A., Angelini A. (2014). Catalysis for the Valorization of Exhaust Carbon: From CO_2_ to Chemicals, Materials, and Fuels. Technological Use of CO_2_. Chem. Rev..

[3] Tang B., Xiao F.-X. (2022). An overview of solar-driven photoelectrochemical CO_2_ conversion to chemical fuels. ACS Catal..

[4] Cheon J., Yang J.Y., Koper M., Ishitani O. (2022). From Pollutant to Chemical Feedstock: Valorizing Carbon Dioxide through Photo-and Electrochemical Processes. Acc. Chem. Res..

[5] Chen C., Jin B.-K. (2012). FTIR Spectroelectrochemistry Study on the Reduction of CO_2_ at a Gold Electrode Interface. Chin. J. Inorg. Chem..

[6] Hartl F., Aarnts M.P., Peelen K. (1996). Infrared Spectroelectrochemical Investigation of Carbon Dioxide Reduction Mediated by the Anion [Ru (SnPh_3_)(Co)_2_(iPr-DAB)]-(iPr-DAB= N,N′-Diisopropyl-1,4-diaza-1,3-butadiene). Coll. Czech. Chem. Comm..

[7] Breelove B.K., Takayama D., Ito T. (2004). Synthesis of an electron pooling ligand based on triruthenium clusters and electrochemical reduction of CO_2_ by its zinc(II)complex. Chem. Lett..

[8] Behnke S.L., Manesis A.C., Shafaat H.S. (2018). Spectroelectrochemical investigations of nickel cyclam indicate different reaction mechanisms for electrocatalytic CO_2_ and H+ reduction. Dalton Trans..

[9] Polyansky D.E., Grills D.C., Ertem M.Z., Ngo K.T., Fujita E. (2022). Role of Bimetallic Interactions in the Enhancement of Catalytic CO_2_ Reduction by a Macrocyclic Cobalt Catalyst. ACS Catal..

[10] Johnson F.P.A., George M.W., Hartl F., Turner J.J. (1996). Electrocatalytic Reduction of CO_2_ Using the Complexes [Re (bpy)(CO)_3_L] n (n=+ 1, L= P (OEt)_3_, CH_3_CN; n= 0, L= Cl-, Otf-; bpy= 2,2‘-Bipyridine; Otf-= CF_3_SO_3_) as Catalyst Precursors: Infrared Spectroelectrochemical Investigation. Organometallics.

[11] Neri G., Donaldsonb P.M., Cowan A.J. (2019). In situ study of the low overpotential “dimer pathway” for electrocatalytic carbon dioxide reduction by manganese carbonyl complexes. Phys. Chem. Chem. Phys..

[12] Filippi J., Rotundo L., Gobetto R., Miller H.A., Nervi C., Lavacchi A., Vizza F. (2021). Turning manganese into gold: Efficient electrochemical CO_2_ reduction by a fac-Mn (apbpy)(CO)_3_Br complex in a gas–liquid interface flow cell. Chem. Eng. J..

[13] Rotundo L., Filippi J., Gobetto R., Miller H.A., Rocca R., Nervi C., Vizza F. (2019). Electrochemical CO_2_ reduction in water at carbon cloth electrodes functionalized with a fac-Mn (apbpy)(CO)_3_ Br complex. Chem. Commun..

[14] Rotundo L., Garino C., Priola E., Sassone D., Rao H., Ma B., Robert M., Fiedler J., Gobetto R., Nervi C. (2019). Electrochemical and photochemical reduction of CO_2_ catalyzed by Re(I)complexes carrying local proton sources. Organometallics.

[15] Sun C., Rotundo L., Garino C., Nencini L., Yoon S.S., Gobetto R., Nervi C. (2017). Electrochemical CO_2_ reduction at glassy carbon electrodes functionalized by MnI and ReI organometallic complexes. ChemPhysChem.

[16] Rotundo L., Azzi E., Deagostino A., Garino C., Nencini L., Priola E., Quagliotto P., Rocca R., Gobetto R., Nervi C. (2019). Electronic effects of substituents on fac-M (bpy-R)(CO)_3_(M = Mn, Re) complexes for homogeneous CO_2_ electroreduction. Front. Chem..

[17] Bourrez M., Molton F., Chardon-Noblat S., Deronzier A. (2011). [Mn (bipyridyl)(CO)_3_Br]: An abundant metal carbonyl complex as efficient electrocatalyst for CO_2_ reduction. Angew. Chem. Int. Ed..

[18] Franco F., Cometto C., Garino C., Minero C., Sordello F., Nervi C., Gobetto R. (2015). Photo-and Electrocatalytic Reduction of CO_2_ by [Re (CO)_3_{α,α′-Diimine-(4-piperidinyl-1,8-naphthalimide)}Cl] Complexes. Eur. J. Inorg. Chem..

[19] Taylor J.O., Neri G., Banerji L., Cowan A.J., Hartl F. (2020). Strong impact of intramolecular hydrogen bonding on the cathodic path of [Re (3,3′-dihydroxy-2, 2′-bipyridine)(CO)_3_Cl] and catalytic reduction of carbon dioxide. Inorg. Chem..

[20] Madsen M.R., Rønne M.H., Heuschen M., Golo D., Ahlquist M.S.G., Skrydstrup T., Pedersen S.U., Daasbjerg K. (2021). Promoting selective generation of formic acid from CO_2_ using Mn (bpy)(CO)_3_Br as electrocatalyst and triethylamine/isopropanol as additives. J. Am. Chem. Soc..

[21] Franco F., Cometto C., Nencini L., Barolo C., Sordello F., Minero C., Fiedler J., Robert M., Gobetto R., Nervi C. (2017). Local proton source in electrocatalytic CO_2_ reduction with [Mn (bpy–R)(CO)_3_Br] complexes. Chem. Eur. J..

[22] Rønne M.H., Cho D., Madsen M.R., Jakobsen J.B., Eom S., Escoudé É., Hammershøj H.C.D., Nielsen D.U., Pedersen S.U., Baik M.-H. (2020). Ligand-controlled product selectivity in electrochemical carbon dioxide reduction using manganese bipyridine catalysts. J. Am. Chem. Soc..

[23] Rønne M.H., Madsen M.R., Skrydstrup T., Pedersen S.U., Daasbjerg K. (2021). Mechanistic Elucidation of Dimer Formation and Strategies for Its Suppression in Electrochemical Reduction of *Fac*-Mn (bpy)(CO)_3_Br. ChemElectroChem.

[24] Madsen M.R., Jakobsen J.B., Rønne M.H., Liang H., Hammershøj H.C.D., Nørby P., Pedersen S.U., Skrydstrup T., Daasbjerg K. (2020). Evaluation of the electrocatalytic reduction of carbon dioxide using rhenium and ruthenium bipyridine catalysts bearing pendant amines in the secondary coordination sphere. Organometallics.

[25] Stor G.J., Hartl F., van Outersterp J.W.M., Stufkens D.J. (1995). Spectroelectrochemical (IR, UV/Vis) Determination of the Reduction Pathways for a Series of [Re(CO)_3_(.alpha.-diimine)L′]0/+ (L′ = Halide, OTf-, THF, MeCN, n-PrCN, PPh3, P(OMe)_3_) Complexes. Organometallics.

[26] Sampson M.D., Nguyen A.D., Grice K.A., Moore C.E., Rheingold A.L., Kubiak C.P. (2014). Manganese catalysts with bulky bipyridine ligands for the electrocatalytic reduction of carbon dioxide: Eliminating dimerization and altering catalysis. J. Am. Chem. Soc..

[27] Machan C.W., Sampson M.D., Chabolla S.A., Dang T., Kubiak C.P. (2014). Developing a Mechanistic Understanding of Molecular Electrocatalysts for CO_2_ Reduction using Infrared Spectroelectrochemistry. Organometallics.

[28] Clark M.L., Grice K.A., Moore C.E., Rheingold A.L., Kubiak C.P. (2014). Reduction by M (bpy-R)(CO)_4_ (M = Mo, W; R = H, tBu) complexes. Electrochemical, spectroscopic, and computational studies and comparison with group 7 catalysts. Chem. Sci..

[29] Cheng S.C., Blaine C.A., Hill M.G., Mann K.R. (1996). Electrochemical and IR spectroelectrochemical studies of the electrocatalytic reduction of carbon dioxide by [Ir_2_(dimen)_4_]^2+^ (dimen = 1,8-Diisocyanomenthane). Inorg. Chem..

[30] Johansson O., Borgström M., Lomoth R., Palmblad M., Bergquist J., Hammarström L., Sun L., Åkermark B. (2003). Electron Donor–Acceptor Dyads Based on Ruthenium (II) Bipyridine and Terpyridine Complexes Bound to Naphthalenediimide. Inorg. Chem..

[31] Krejčik M., Daněk M., Hartl F. (1991). Simple construction of an infrared optically transparent thin-layer electrochemical cell: Applications to the redox reactions of ferrocene, Mn_2_(CO)_10_ and Mn(CO)_3_(3,5-di-t-butyl-catecholate)^−^. J. Electroanal. Chem. Interfacial Electrochem..

[32] Kaim W., Fiedler J. (2009). Spectroelectrochemistry: The best of two worlds. Chem. Soc. Rev..

[33] Frisch M.J., Trucks G.W., Schlegel H.B., Scuseria G.E., Robb M.A., Cheeseman J.R., Scalmani G., Barone V., Mennucci B., Petersson G.A. (2009). Gaussian 09.

[34] Miertuš S., Scrocco E., Tomasi J. (1981). Electrostatic interaction of a solute with a continuum. A direct utilizaion of AB initio molecular potentials for the prevision of solvent effects. J. Chem. Phys..

[35] Cossi M., Scalmani G., Rega N., Barone V. (2002). New developments in the polarizable continuum model for quantum mechanical and classical calculations on molecules in solution. J. Chem. Phys..

[36] Becke A.D. (1993). Density-functional thermochemistry. I. The effect of the exchange-only gradient correction. J. Chem. Phys..

[37] Lee C., Yang W., Parr R.G. (1988). Development of the Colle-Salvetti correlation-energy formula into a functional of the electron density. Phys. Rev. B Condens. Matter.

[38] Weigend F. (2006). Accurate Coulomb-fitting basis sets for H to Rn. Phys. Chem. Chem. Phys..

[39] Weigend F., Ahlrichs R. (2005). Balanced basis sets of split valence, triple zeta valence and quadruple zeta valence quality for H to Rn: Design and assessment of accuracy. Phys. Chem. Chem. Phys..

[40] Grimme S., Ehrlich S., Goerigk L. (2011). Effect of the damping function in dispersion corrected density functional theory. J. Comput. Chem..

