# Supplementary material for: New Spectroelectrochemical Insights into Manganese and Rhenium Bipyridine Complexes as Catalysts for the Electrochemical Reduction of Carbon Dioxide
Source: Molecules. 2023 Nov 10;28(22):7535. doi: 10.3390/molecules28227535 (PMC10673266; doi:10.3390/molecules28227535)
Supplement: Supplementary file 1 [file molecules-28-07535-s001.zip › molecules-2639163-supplementary.pdf]

Supporting Information

New Spectroelectrochemical Insights in Mn and Re bipyridine Complexes as Catalysts for the Electrochemical Reduction of CO_2

Alice Barbero [1], Laura Rotundo [1,2], Chiara Reviglio [1], Roberto Gobetto [1], Romana Sokolova [3], Jan Fiedler [3] and Carlo Nervi [1*]

Table of Content

Figure S1. Spectroelectrochemistry of **1** in MeCN under CO_2: reduction behind the second reduction peak (-2.1 V *vs*. Fc/Fc$^+$) … 2

Figure S2. Spectroelectrochemistry of **3** in MeCN under CO_2: first reduction … 3

Figure S3. Spectroelectrochemistry of **3** in MeCN under CO_2: second reduction … 4

Figure S4. Spectroelectrochemistry of **1** in DMA: first reduction … 5

Figure S5. Spectroelectrochemistry of **1** in DMA: second reduction … 5

Figure S6. Spectroelectrochemistry of **2** in DMA: first reduction … 6

Figure S7. Spectroelectrochemistry of **2** in DMA: second reduction … 6

Figure S8. Spectroelectrochemistry of **3** in DMA: first reduction … 7

Figure S9. Spectroelectrochemistry of **3** in DMA: second reduction … 7

Figure S10. Spectroelectrochemistry of **4** in DMA: two-electron reduction … 8

DFT optimized structures of **1**, **1**$^-$, **2**, and **2**$^-$ … 8

Figure S11. ^{1}H NMR spectra of the ligand 4-(4-aminophenyl)-2,2′-bipyridine in $(CD_3)_2CO$ ….8

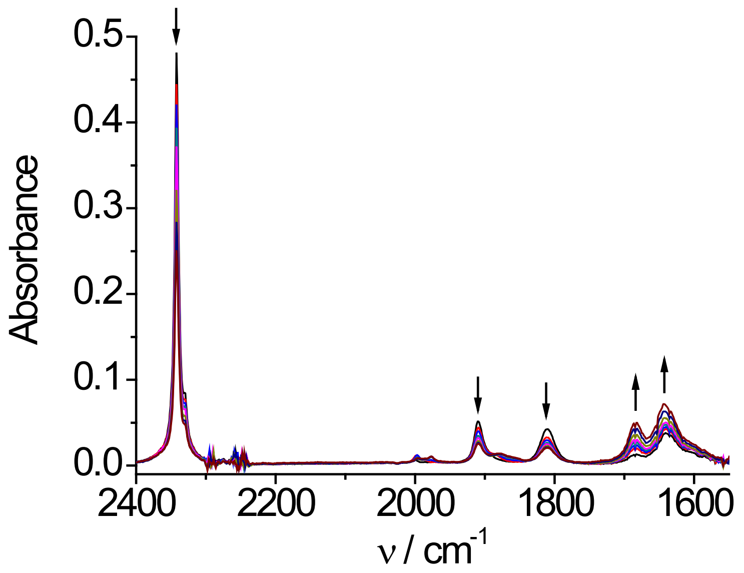

Figure S1. IR **s**pectroelectrochemistry of **1** in MeCN / 0.1 $TBAPF_6$ under CO_2: reduction behind the second reduction peak (-2.1 V *vs*. Fc/Fc^+). Noise between ≈2250 and 2300 cm^{-1} is due to solvent absorptions and subsequent background subtraction.

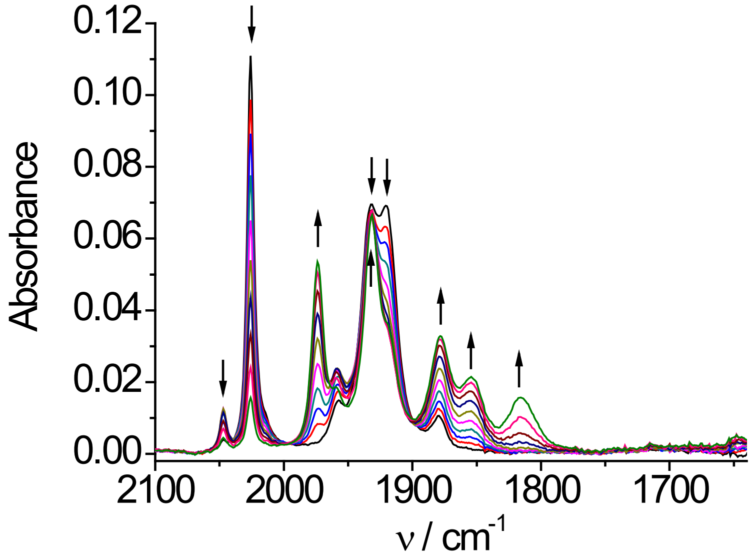

Figure S2. IR spectroelectrochemistry of **3** in MeCN / 0.1 M $TBAPF_6$ under CO_2: reduction at the first reduction peak (last spectra show a partial formation of the 2e reduction product)

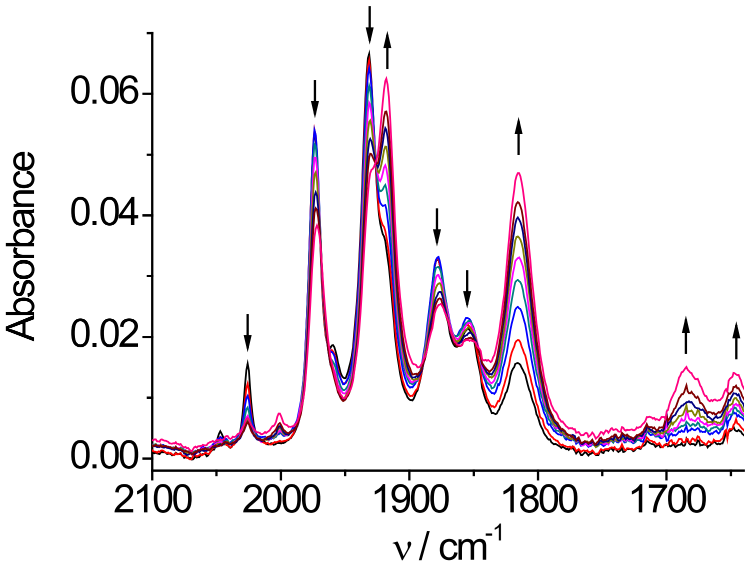

Figure S3. IR spectroelectrochemical response on the second reduction of **3** in MeCN / 0.1 M TBAPF$_6$ under CO_2 (and still finishing first reduction in the beginning).

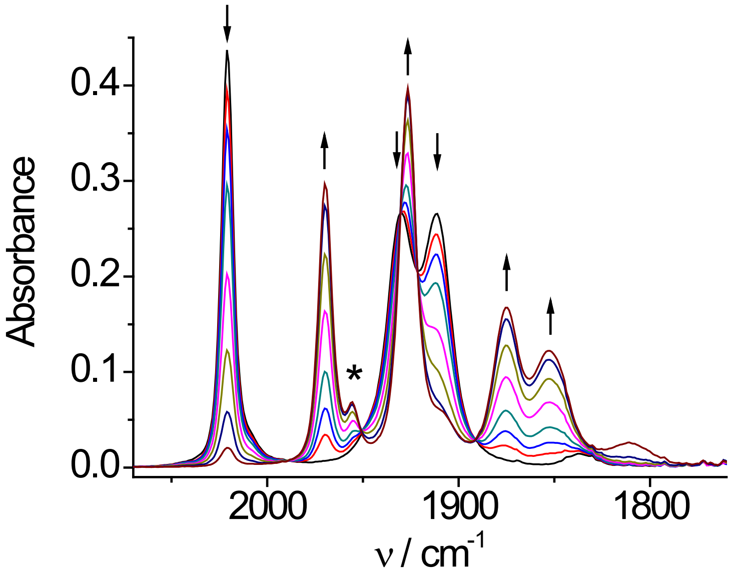

Figure S4. IR spectroelectrochemical response on the first reduction of **1** in DMA / 0.1 M TBAPF$_6$ (* = unidentified side-product)

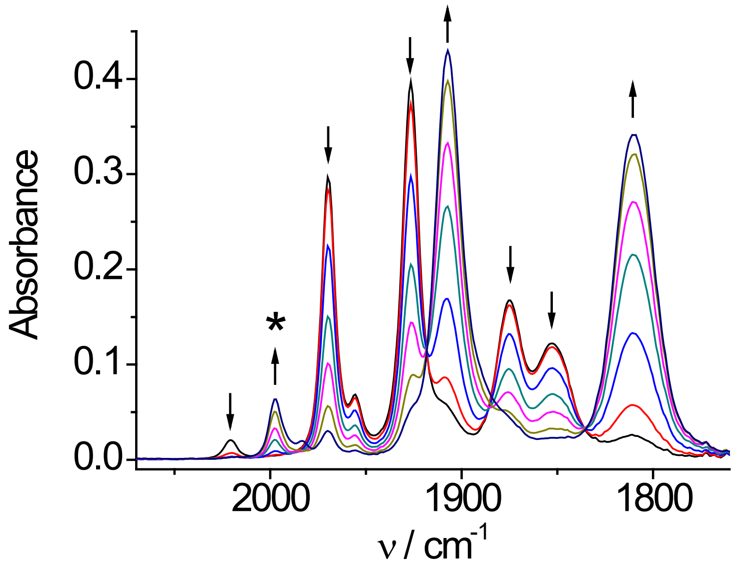

Figure S5. IR spectroelectrochemical response on the second reduction of **1** in DMA / 0.1 M $TBAPF_6$ (* = unidentified side-product)

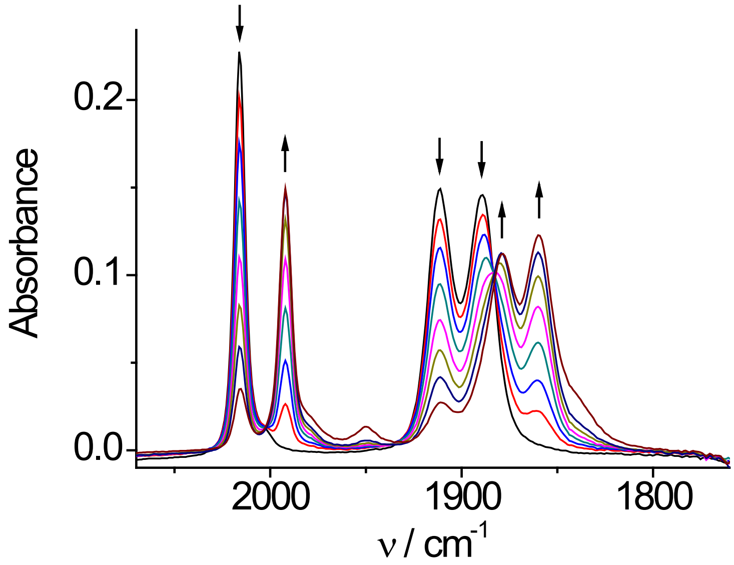

Figure S6. IR spectroelectrochemical response on the first reduction of **2** in DMA / 0.1 M $TBAPF_6$.

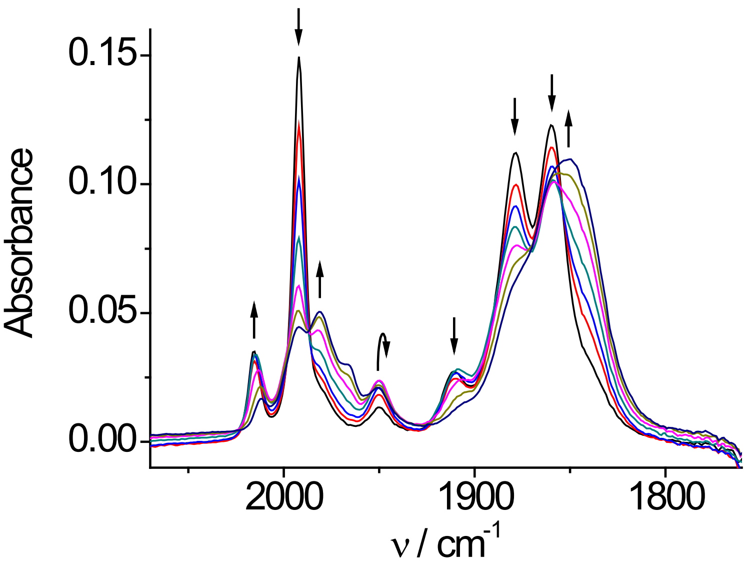

Figure S7. IR spectroelectrochemical response on the second reduction of **2** in DMA / 0.1 M $TBAPF_6$.

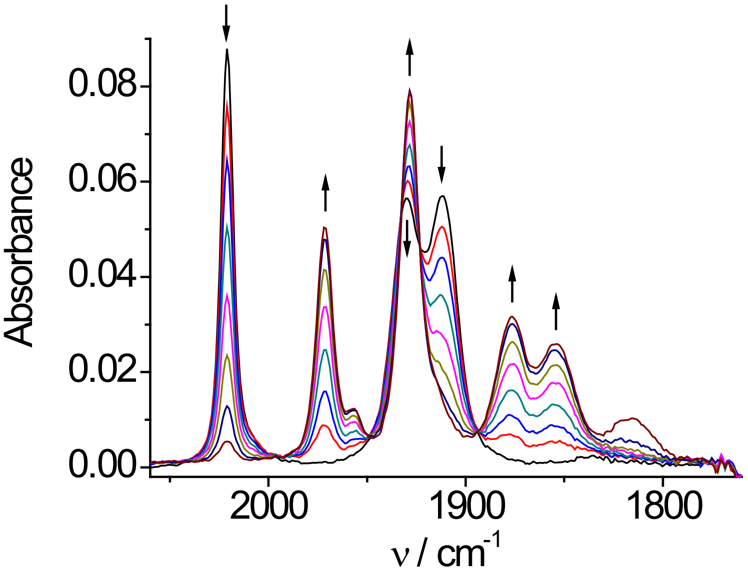

Figure S8. IR spectroelectrochemical response on the first reduction of **3** in DMA / 0.1 M $TBAPF_6$.

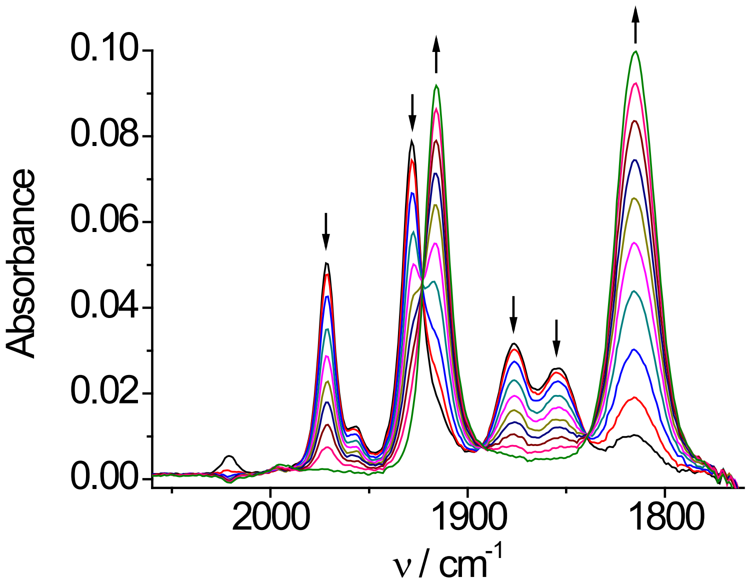

Figure S9. IR spectroelectrochemical response on the second reduction of **3** in DMA / 0.1 M $TBAPF_6$.

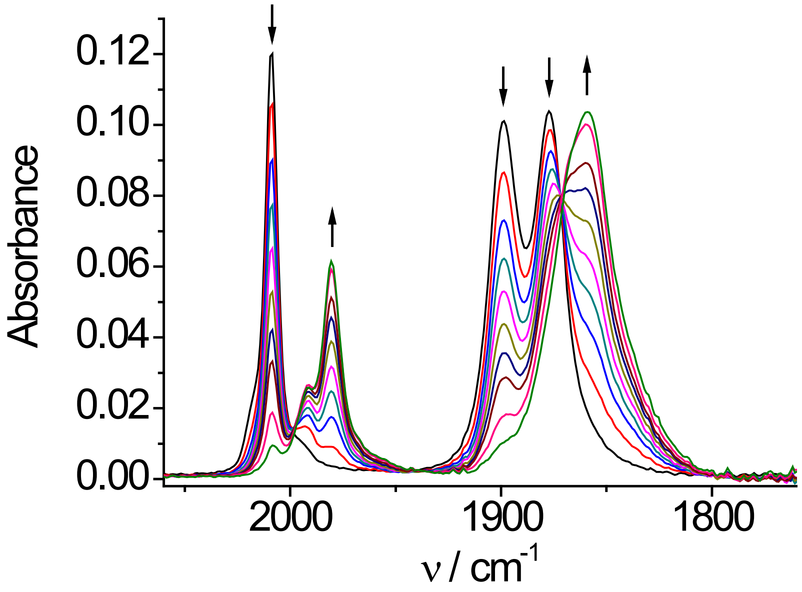

Figure S10. IR spectroelectrochemical response on reduction of **4** in DMA / 0.1 M $TBAPF_6$ (two-electron CV peak).

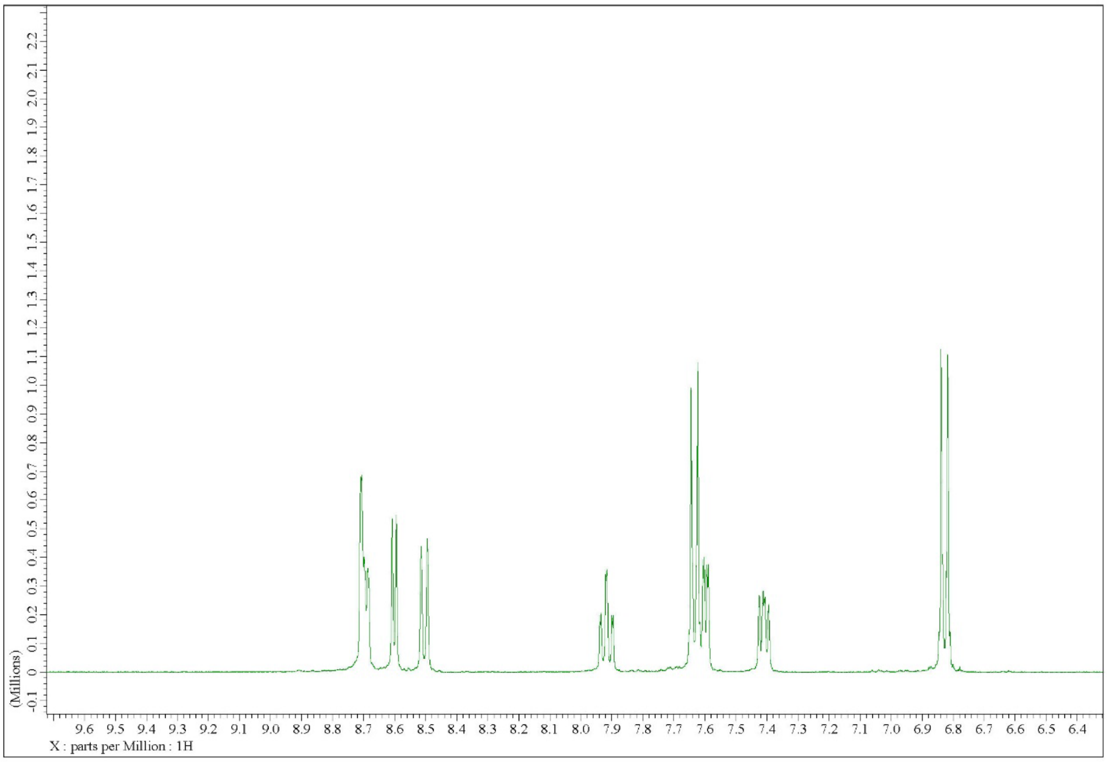

Figure S11. ^{1}H NMR spectra of the ligand 4-(4-aminophenyl)-2,2‘-bipyridine in $(CD_3)_2CO$.

DFT optimized geometry of **1**.

```
C 1.89971300 -1.62870300 0.27796700
C 2.54764100 -0.38073500 0.17760600
C 1.70578700 0.74766600 0.17726600
C 0.32213200 0.60931100 0.26423600
C 0.51709300 -1.69238400 0.36490200
C -0.60768000 1.75420100 0.27591900
C -0.19744200 3.09006400 0.19764400
C -1.15362700 4.10219100 0.22122600
H -0.84941900 5.14855100 0.16090600
C -2.50066800 3.75268300 0.32403100
C -2.83663300 2.40358600 0.39594400
H 2.46217100 -2.56153200 0.26586800
H 0.01965200 -2.65966600 0.43416100
H -3.28758600 4.50721200 0.34786500
H -3.87946200 2.09710200 0.47368500
N -0.27063900 -0.60541900 0.35448500
N -1.91809500 1.42623000 0.37032100
C -2.49831800 -2.40331400 0.26947000
O -2.60688100 -3.54484300 0.17007300
C -4.13347900 -0.40873400 0.28502200
O -5.27548400 -0.29486200 0.19579200
C -2.34825200 -0.62933400 2.20569800
O -2.35325700 -0.64417900 3.35753700
H 0.85953000 3.33858100 0.11832800
H 2.13788900 1.74434300 0.13164500
C 4.00926100 -0.25928400 0.08104100
C 4.62029100 0.88539000 -0.47445000
C 4.86463500 -1.28503000 0.53729900
C 5.99873100 1.00311300 -0.57345900
H 4.00431600 1.69541100 -0.86933600
C 6.24491500 -1.17706800 0.45381600
H 4.44365600 -2.18274600 0.99357200
```

```
C 6.85023100 -0.02662700 -0.10635700
H 6.43701800 1.89699100 -1.02369800
H 6.87713800 -1.98472600 0.83039200
N 8.20879400 0.10023800 -0.16261600
H 8.78709500 -0.71865700 -0.02148900
H 8.61261900 0.82824600 -0.73866900
Br -2.24296200 -0.50069000 -2.20080500
Mn -2.32809500 -0.59772700 0.40293900
```

DFT optimized geometry of **1**$^-$.

```
C 1.90859000 -1.62242900 0.32384700
C 2.55830200 -0.35380800 0.17982300
C 1.73071000 0.76192400 0.15629000
C 0.32386200 0.64929700 0.25557600
C 0.53401800 -1.67643100 0.42164400
C -0.57940900 1.75862800 0.25431300
C -0.19306500 3.12083200 0.14177700
C -1.14246300 4.11958200 0.16267500
H -0.84540800 5.16682500 0.07416800
C -2.50963300 3.76427500 0.30295600
C -2.83040300 2.42315900 0.41053500
H 2.47464800 -2.55321000 0.32537900
H 0.03737300 -2.64314500 0.52348100
H -3.29889900 4.51654100 0.32438500
H -3.87247000 2.11747600 0.51924500
N -0.27205700 -0.59451100 0.38480400
N -1.91883900 1.42899200 0.38346400
C -2.46988200 -2.37507300 0.39035200
O -2.57923700 -3.52163100 0.30405200
C -4.10444500 -0.38070700 0.38672200
O -5.25039700 -0.26547500 0.29889700
C -2.31365100 -0.57840300 2.29627100
O -2.29418900 -0.56232100 3.45249900
H 0.86278300 3.37161200 0.03716200
H 2.16750900 1.75709200 0.08483300
C 4.02565600 -0.24531400 0.07217200
C 4.64717500 0.86039500 -0.54614900
C 4.87832800 -1.24746400 0.57989700
C 6.02944400 0.96730900 -0.64510300
H 4.03311500 1.64852300 -0.98689800
C 6.26366100 -1.15106500 0.48968200
H 4.45096100 -2.11869100 1.08000200
C 6.87498200 -0.03781000 -0.12511300
H 6.47237700 1.83460100 -1.14196100
H 6.89081000 -1.94437000 0.90517300
N 8.24863300 0.08815500 -0.17585900
H 8.79086400 -0.76354100 -0.08167400
H 8.62467100 0.72689600 -0.86785700
Mn -2.30459900 -0.57286700 0.51809700
Br -2.35364600 -0.59998200 -2.38249500
```

DFT optimized geometry of **2**.

```
C 2.19969000 -1.60750000 0.01526500
C 2.88960700 -0.37713400 0.03821300
C 2.08296500 0.77717700 0.08227800
C 0.69363900 0.68596300 0.09445800
C 0.81515100 -1.63096000 0.03414900
C -0.18870800 1.87109700 0.14939500
C 0.28925000 3.18602500 0.18013000
C -0.61301500 4.24502000 0.24045800
H -0.25047100 5.27402300 0.26449300
C -1.98048800 3.96751000 0.26961100
C -2.39095100 2.63931200 0.23368600
H 2.73229200 -2.55593300 -0.04035300
H 0.27804700 -2.57905900 0.00890000
H -2.72562200 4.76207700 0.31731600
H -3.44895400 2.37867600 0.25069100
N 0.06296000 -0.51631500 0.07128500
N -1.52122100 1.61640800 0.17498400
Re -2.12968500 -0.49409800 0.02269400
C -2.39665400 -2.39128900 -0.22686000
O -2.50867700 -3.53335900 -0.38733400
C -4.03541600 -0.19995400 -0.11513000
O -5.16892300 0.02397100 -0.20496200
C -2.27098800 -0.69645900 1.92429500
```

O -2.34812000 -0.81260600 3.07719100
Cl -1.78983000 -0.10735700 -2.45577600
H 1.35895600 3.38442600 0.15572700
H 2.54999800 1.75723200 0.13131000
C 4.35529500 -0.29744900 0.01993500
C 5.02765500 0.87461600 -0.39073700
C 5.15710100 -1.39214100 0.41083000
C 6.41133700 0.95416400 -0.41337100
H 4.45764300 1.74067400 -0.73159000
C 6.54165000 -1.32429100 0.40291500
H 4.68863200 -2.31543500 0.75562600
C 7.20815300 -0.14512100 -0.01055500
H 6.89804100 1.87191600 -0.75170900
H 7.13030900 -2.18620100 0.72574100
N 8.56834900 -0.06046500 0.00720800
H 9.12107800 -0.90040000 0.12223400
H 9.02706200 0.71743800 -0.44954700

DFT optimized geometry of $\mathbf{2}^{-}$.

C 2.19905900 -1.61199700 -0.00904100
C 2.88562000 -0.35336900 0.02591600
C 2.09258100 0.78434700 0.07952500
C 0.67636600 0.71458800 0.08815300
C 0.82095200 -1.63146200 0.00413900
C -0.18127700 1.85715200 0.14727800
C 0.27456600 3.20498700 0.19553400
C -0.62235900 4.24767700 0.25566800
H -0.26557300 5.27935000 0.29199800
C -2.01496800 3.96399200 0.26938200
C -2.40995000 2.64042800 0.21935400
H 2.74125000 -2.55401200 -0.07961200
H 0.28596100 -2.58252800 -0.03249300
H -2.76291900 4.75599600 0.31556300
H -3.46948400 2.37756500 0.22557200
N 0.04918500 -0.52460000 0.04941900
N -1.54777200 1.60354900 0.15828600
Re -2.12428300 -0.49446900 0.03491900
C -2.37652100 -2.39585400 -0.20787000
O -2.48164000 -3.54380400 -0.36231200
C -4.03063200 -0.19754800 -0.09887800
O -5.16854000 0.02733200 -0.18570900
C -2.25690300 -0.68825500 1.92562300
O -2.32840500 -0.79887800 3.08360300
Cl -1.83361200 -0.14437100 -2.50810100
H 1.34516400 3.40856800 0.18288100
H 2.56321300 1.76417100 0.14132100
C 4.35929400 -0.28025100 0.01205000
C 5.04371800 0.86210200 -0.45438500
C 5.15463400 -1.35461000 0.46144500
C 6.43158400 0.93567200 -0.46356200
H 4.47655200 1.70870300 -0.84689500
C 6.54485200 -1.29241900 0.46057000
H 4.67625200 -2.25809600 0.84436600
C 7.21924900 -0.14212900 -0.00119100
H 6.92508500 1.83405800 -0.84385500
H 7.12599500 -2.14272400 0.82732900
N 8.59580200 -0.05355700 0.03939900
H 9.11160600 -0.92562400 0.07957500
H 9.02849000 0.64078900 -0.55958900
